# Informal caregiver well-being during and after patients’ treatment with adjuvant chemotherapy for colon cancer: a prospective, exploratory study

**DOI:** 10.1007/s00520-020-05738-w

**Published:** 2020-09-15

**Authors:** S. M. C. H. Langenberg, H. Poort, A. N. M. Wymenga, J. W. de Groot, E. W. Muller, W. T. A. van der Graaf, J. B. Prins, C. M. L. van Herpen

**Affiliations:** 1 Department of Medical Oncology, Radboud University Medical Center, P.O. Box 9101, 6500 HB Nijmegen, The Netherlands; 2 Dana-Farber Cancer Institute, Department of Psychosocial Oncology and Palliative Care, Boston, MA USA; 3 Harvard Medical School, Boston, MA USA; 4 Department of Internal Medicine, Medisch Spectrum Twente, Enschede, The Netherlands; 5 Department of Internal Medicine, Isala, Zwolle, The Netherlands; 6 Department of Internal Medicine, Slingeland Ziekenhuis, Doetinchem, The Netherlands; 7 Department of Medical Oncology, Netherlands Cancer Institute, Amsterdam, The Netherlands; 8 Department of Medical Psychology, Radboud University Medical Center, Nijmegen, The Netherlands

**Keywords:** Caregiver, Colon cancer, Burden, Distress

## Abstract

**Introduction:**

Caring for a significant other during cancer treatment can be demanding. Little is known about the well-being of informal caregivers of patients with colon cancer. This study aims to examine informal caregiver well-being during adjuvant chemotherapy for colon cancer.

**Material and methods:**

This exploratory longitudinal, prospective study measured the course of informal caregiver burden (Self-Perceived Pressure of Informal Care), distress (Hospital Anxiety and Depression Scale), health-related quality of life (RAND-36), marital satisfaction (Maudsley Marital Questionnaire), social support (Social Support List – Discrepancies), fatigue (Abbreviated Fatigue Questionnaire), and self-esteem (Caregiver Reaction Assessment) before (T0), during (T1), and after (T2) patients’ treatment.

**Results:**

Baseline data of 60 out of 76 eligible dyads (79%) were analyzed. Mean levels of informal caregiver burden and distress improved significantly over time, as did their health-related quality of life and perceived social support. At baseline, 30% and 26.7% of informal caregivers reported moderate-to-high levels of burden and clinically relevant levels of distress, respectively, which changed to 20% and 18.8% at T2. Informal caregiver burden and distress at baseline were the strongest predictors of informal caregiver burden and distress during and following patients’ treatment, respectively.

**Conclusion:**

When informal caregivers and patients experience problems before start of adjuvant chemotherapy, problems seem to improve over time. Approximately 20% of informal caregivers remain burdened and distressed after patients’ end of treatment. Paying attention to baseline distress and burden seems indicated, as these were strong predictors of informal caregivers’ well-being during and after treatment.

**Electronic supplementary material:**

The online version of this article (10.1007/s00520-020-05738-w) contains supplementary material, which is available to authorized users.

## Introduction

Colon cancer is a common cancer among men and women [1]. In 2018, colon cancer accounted for 6% of all new cancer cases and 551,269 people died as a consequence of colon cancer worldwide [1]. In 2018, 9555 people were newly diagnosed with colon cancer in The Netherlands [2]. After diagnosis and staging, treatment plans are made according to (inter)national guidelines. When a patient is diagnosed with high-risk stage II or III colon cancer, surgical resection alone results in a 5-year survival rate of 60–80% for stage II and 30–60% for stage III disease [3]. To further improve survival, patients receive adjuvant chemotherapy containing fluoropyrimidines and oxaliplatin (i.e., 5FU/leucovorin with oxaliplatin (FOLFOX) or capecitabine with oxaliplatin (CAPOX)), or capecitabine monotherapy when oxaliplatin is contra-indicated [4, 5]. Common side effects of this regimen are nausea, vomiting, hand-foot syndrome, and sensory, peripheral neuropathy. Being diagnosed with cancer and receiving cancer treatment impact the patient and can cause psychological distress [6].

After a cancer diagnosis and during cancer treatment, support by a significant other is indispensable. Informal caregivers support patients both practically and emotionally [6, 7]. These tasks can cause burden and distress, which may impact an informal caregiver’s ability to support the patient during treatment [8, 9]. Informal caregiver burden can be experienced on several dimensions and be related to emotional, social, physical, spiritual functioning, and/or practical and financial problems [7]. The National Cancer Institute defined distress as “emotional, social, spiritual or physical pain or suffering that may cause a person to feel sad, afraid, depressed, anxious or lonely” [10]. Previous studies identified burden and distress as important problems for informal caregivers [6, 9]. Patients and informal caregivers cope with cancer as a dyad and informal caregivers’ distress may sometimes exceed patients’ distress [11, 12]. Informal caregiver burden and distress are interrelated and share risk factors, such as female gender, younger age, and perceived patient distress [7, 9]. Importantly, informal caregiver burden and distress may also negatively influence the informal caregivers’ physical health and social functioning [6–9]. Little is known about the course of well-being of informal caregivers of patients treated with adjuvant chemotherapy for colon cancer.

This prospective, observational study had four exploratory aims. First, we aimed to examine the course of informal caregiver well-being. Second, we intended to explore the clinically relevant levels of informal caregiver burden and distress. Third, we aimed to identify baseline risk factors for higher informal caregiver burden and distress during and after a patient’s treatment. Fourth, we explored the association between informal caregiver burden and distress and patient distress before, during, and after adjuvant chemotherapy.

## Material and methods

### Setting and participants

We conducted a prospective, observational study between October 2013 and September 2017 in four hospitals in The Netherlands. We recruited patients aged 18 or older, proficient in Dutch, who were scheduled to receive adjuvant chemotherapy (CAPOX or capecitabine monotherapy) after surgery for colon cancer, and their informal caregivers, for participation in this study.

### Procedure

The attending physician or the nurse practitioner approached the patient and their informal caregiver for study participation after informing them about starting adjuvant chemotherapy. Written informed consents were obtained from both patient and informal caregiver. The study included completion of paper-and-pencil questionnaires at home at three time points: (1) baseline, before starting adjuvant chemotherapy (T0), (2) between the second and third cycle (T1), and (3) 3 months after ending adjuvant chemotherapy (T2). We asked patients and informal caregivers to complete questionnaires separately. The study was approved by the local medical ethics committee of the Radboud University Medical Center (registration number 2013/393).

### Measures

#### Sociodemographic and clinical factors

Patients and informal caregivers self-reported their age, sex, level of education (categorized using the International Standard Classification of Education), and employment (paid work, housekeeper, disablement insurance act, retired, volunteer, study) at baseline. Informal caregivers completed a general questionnaire on the nature of their relation to the patient (partner, child, sibling, friend), whether they lived together with the patient (yes/no) and the extent of their caregiving tasks (hours of caring, independency of the patient, caring for more than patient alone), as well as their needs during caring for their significant other (practical and/or emotional support from social support system and/or professionals, information services from hospital and/or general practitioner, and/or better communication with physician/nurses/general practitioner). Furthermore, we inquired whether the treatment side effects of cancer treatment had a negative influence on informal caregiver well-being (no/yes, somewhat/yes).

Patients self-reported whether they had a colostomy after surgery, the total number of cycles of adjuvant chemotherapy, and experienced toxicity (T1 and T2). Patients also reported what sort of complications they had after surgery. The attending medical oncologist provided information which adjuvant chemotherapy (CAPOX versus capecitabine monotherapy) was prescribed.

We used the Self-administered Comorbidity Questionnaire (SCQ) to assess 14 common medical conditions in both patients and informal caregivers, and additional comorbidities could also be reported [13]. For each condition, patients and informal caregivers indicated whether it was present, being treated, or imposed functional limitations. For the present study, we used data on whether a comorbidity was present and causing functional limitations.

#### Informal caregiver well-being

Informal caregivers completed the 9-item Self-Perceived Pressure from Informal Care (SPPIC) [14]. Items are scored on a 5-point scale ranging from 0 (no!) to 5 (yes!). We dichotomized scores to 0 (“no!” and “no”) and 1 (“yes!,” “yes,” and “more or less”). Total scores range from 0 to 9, and higher scores indicate higher levels of perceived informal caregiver burden. In accordance with other studies [15–17], we classified informal caregivers into low (0–3), moderate (4–6), and high levels of burden (7–9) based on their total score. Patients and informal caregivers each completed the Hospital Anxiety and Depression Scale (HADS) to measure distress [18]. The HADS consists of 14 items assessing symptoms of anxiety and depression during the past week. Items are scored on a 4-point Likert scale ranging from 0 to 3. Total scores range from 0 to 42, and higher scores indicate more distress. We used the 36-item RAND-36 Health Survey to assess functional status, well-being, and general health [19]. Scores on each of the eight subscales are transformed into a range from 0 to 100; higher scores indicate higher levels of functioning, well-being, and general health. The Social Support List – Discrepancies (SSL-D) is a 34-item questionnaire assessing discrepancies between an individual’s need for social support and their perceived social support [20, 21]. The questionnaire assesses six types of social support, namely emotional interactions, problem-focused emotional support, esteem support, instrumental interactions, social companionship, and informational support. The score on every item is transformed into a sum score ranging from 34 to 136. Higher scores indicate more unmet needs for social support. We used the “marital satisfaction” subscale of the Maudsley Marital Questionnaire (MMQ) to assess marital satisfaction [22]. This is a 10-item questionnaire, answered on a 9-point scale (0–8), ranging from 0 to 80. Higher scores indicate decreased marital satisfaction. We instructed participants to complete this questionnaire if they had a partner relationship. We administered the Abbreviated Fatigue Questionnaire (AFQ), a validated 4-item questionnaire to measure fatigue [23, 24]. The AFQ is an abbreviated version of the subscale fatigue severity of the Checklist Individual Strength. Items are scored on a 7-point scale ranging from “no, that’s not correct” (score 1) to “yes, that’s correct” (score 7). The total score on the 4-item questionnaire ranges from 4 to 28. Higher scores indicate greater levels of fatigue. Informal caregiver self-esteem was measured by the subscale caregiver self-esteem of the Caregiver Reaction Assessment Scale (CRA) [25]. The subscale consists of 7 items, scored on a 5-point Likert scale. The total score reflects a mean score of 7 items and ranges between 1 and 5; a higher score indicates more self-esteem.

### Statistical analysis

Descriptive statistics were used for baseline characteristics of the patients and informal caregivers. Chi-square and Fisher’s exact tests were performed to examine the relation between categorical variables. Independent-samples *t* tests and Mann-Whitney *U* tests were used to compare continuous variables. We analyzed informal caregiver burden, distress, health-related quality of life, marital satisfaction, discrepancies in social needs, fatigue, and informal caregiver self-esteem with one-way ANOVA with repeated measures. Next, we applied the same statistical analyses for patient distress, health-related quality of life, marital satisfaction, discrepancies in social needs, and fatigue. We applied the Greenhouse-Geisser correction when the assumption of sphericity was violated. Post hoc tests with Bonferroni’s correction were used to identify which specific means differed. To identify cases of clinically significant distress, we applied a HADS total clinical cutoff score of ≥ 11 to identify patients with clinically significant distress [26] and a cutoff score of ≥ 12 for informal caregivers [27]. To identify possible cases of depression (HADS-D) and anxiety (HADS-A), we used the cutoff score of ≥ 8 [26, 27]. McNemar’s tests were carried out to determine whether the proportion of patients or informal caregivers exceeding the cutoff for clinically relevant distress differed between baseline and T2. We used multiple linear regression to predict informal caregiver burden and distress at T1 and T2 from informal caregiver gender, age, burden, distress, fatigue, and patient distress at baseline (method: enter). For the linear regression analyses, we used the continuous variables of burden, distress, and fatigue. All data analyses were performed using the statistical package for the social sciences (SPSS, version 24.0). Statistical significance was determined based on a two-sided alpha of 0.05.

## Results

Of 76 eligible patients and their informal caregivers, 62 (82%) dyads enrolled in the study, and 14 (18%) declined participation, mostly because participation was considered too burdensome. Of the 62 dyads that provided consent, 2 dyads withdrew consent after enrollment. Thus, T0 was completed by 60 (79%), whereas T1 by 58 (76%) and T2 by 51 dyads (67%). Baseline, caregiving, and treatment characteristics are provided in Table 1. Table 2 displays an overview of what support informal caregivers need before, during, and after a patients’ treatment with adjuvant chemotherapy. When informal caregivers reported they need support, it seemed most needed before starting chemotherapy and focused on practical support and receiving information.

**Table 1:** Characteristics of informal caregivers and patients

	Informal caregivers (*n* = 60)	Patients (*n* = 60)
Age, years (SD)	59.88 (12.72)	63.83 (7.74)
Sex		
Male	25 (41.7%)	34 (56.7%)
Female	35 (58.3%)	26 (43.3%)
Education		
Lower education level (ISCED ≤ 4)	35 (58.3%)	41 (69.5%)
Higher education level (ISCED 5–8)	25 (41.6%)	18 (30.5%)
Missing	0	1
Dependent children (lives with)		
No	43 (71.7%)	
Yes	9 (15.0%)	
Missing	8 (13.3%)	
Employment^a^		
Paid work	23 (38.3%)	19 (31.7%)
Housekeeper	9 (15%)	7 (11.7%)
Disablement insurance act	0 (0%)	7 (11.7%)
Retired	25 (41.7%)	28 (46.7%)
Volunteer work	4 (6.7%)	3 (5%)
Study	2 (3.3%)	0 (0%)
Informal caregiver’s relationship to patient		
Partner	53 (91.4%)	
Child	2 (3.4%)	
Sibling	2 (3.4%)	
Friend	1 (0.8%)	
Missing	2	
Informal caregiver lives with patient		
Yes	53 (89.8%)	
No	6 (10.2%)	
Missing	1	
Providing > 8 h of care		
T0	19/60 (31.9%)	
T1	18/57 (31.6%)	
T2	5/51 (9.9%)	
Patients functioned independently or mostly independently		
T0	51/57 (89.5%)	
T1	51/57 (89.5%)	
T2	48/51 94.2%	
Providing care to more than patient alone		
T0	7/60 (11.9%)	
T1	5/57 (8.8%)	
T2	3/51 (5.9%)	
Negative influence of patients’ side effects on caregiver well-being (measured on T1)		
No	22/53 (41.5%)	
Yes, somewhat	27/53 (50.9%)	
Yes	4/53 (7.5%)	
Colostomy after surgery		
No		52 (86.7%)
Yes		8 (13.3%)
Complications after surgery^a^		
No		28 (46.7%)
Obstipation		8 (13.4%)
Wound leakage/infection		8 (13.4%)
Anastomotic leakage		3 (5.0%)
Bleeding/anemia		2 (3.4%)
Thrombosis		1 (1.7%)
High blood pressure		1 (1.7%)
Urinary catheter		1 (1.7%)
Urinary tract infection		1 (1.7%)
Gastroparesis		2 (3.4%)
Ileus		1 (1.7%)
Cardiac arrest		1 (1.7%)
Stoma retraction		1 (1.7%)
Type of adjuvant chemotherapy		
CAPOX		53 (88.3%)
Capecitabine monotherapy		7 (11.7%)
Number of cycles completed		
8		37 (72.5%)
7		6 (7.8%)
6		4 (7.8%)
5		1 (2%)
4		1 (2%)
3		1 (2%)
2		1 (2%)
1		0 (0%)
Missing		9
Reason for modification treatment (n = 26)		
Discontinuation oxaliplatin only		13/26 (50%)
Discontinuation oxaliplatin and capecitabine		13/26 (50%)
Toxicity 3 months after ending adjuvant treatment		
No		1 (2%)
Yes, suffered from side effect, but disappeared		15 (29.4%)
Yes, suffered from side effects, now barely noticeable		4 (7.8%)
Yes, suffered from side effects and still do		31 (60.8%)
Missing		9
Comorbidities		
0	16 (29.1%)	11 (20.0%)
1–2	34 (61.8%)	35 (63.7%)
3 or more	5 (9.1%)	9 (16.4%)
Missing	5	5
Most common comorbidities		
High blood pressure	15/59 (25.4%)	22/58 (37.9%)
Back pain	15/57 (26.3%)	14/58 (24.1%)
Arthrosis	12/57 (21.1%)	8/58 (13.8%)
Chronic obstructive pulmonary disease	10/57 (17.5%)	7/57 (16.7%)
Diabetes	6/58 (10.3%)	10/58 (17.2%)
Hearth disease	2/57 (3.5%)	11/58 (19%)
Hindrance from comorbidit(y)(ies)		
High blood pressure	1/15 (6.7%)	0/22 (0%)
Back pain	7/15 (46.7%)	6/12 (50%)^c^
Arthrosis	6/11 (54.5%)^b^	1/8 (12.5%)
Chronic obstructive pulmonary disease	5/10 (50%)	1/6 (16.7%)^b^
Diabetes	1/6 (16.7%)	1/9 (11.1%)^b^
Heath disease	0/2 (0%)	2/9 (18.2%)^c^

^a^Multiple answers possible

^b^One missing answer

^c^Two missing answers

**Table 2:** Informal caregivers’ needs for support before, during, and after adjuvant chemotherapy

	Baseline (*n* = 60)	After second Tx cycle (*n* = 57)	3 months after completing Tx (*n* = 51)
No support needed	39 (65.0%)	43 (75.4%)	42 (82.4%)
Practical support			
From social support system	5	3	1
From professional			
Emotional support	8	4	4
From social support system			
From professional	1	0	1
Information services			
From hospital	2	1	2
From general practitioner			
Better communication	6	2	1
With physician	3	1	2
With nurses			
With general practitioner	1	1	2
Other	0	1	1
	1	2	1
	3	2	1

### Course of burden, distress, health-related quality of life, marital satisfaction, discrepancies in social needs, fatigue over time, and informal caregiver self-esteem

Table 3 shows mean scores for informal caregiver well-being over time. Mean scores for informal caregiver burden (*F*[2,94] = 4.465; *p* = 0.014), distress (*F*[1.773,81.574] = 5.497; *p* = 0.008), role emotional limitations (*F*[2,94] = 8.814; *p* < 0.0001), mental health (*F*[2,94] = 4.949; *p* = 0.009), social functioning (*F*[2,98] = 3.985; *p* = 0.022), and discrepancies in social support (*F*[2,66] = 3.466; *p* = 0.037) differed significantly between time points. Post hoc analyses indicated an improvement of all scores over time; i.e., burden, distress, and role emotional limitations decreased, whereas mental health and social functioning increased. Discrepancies in social support decreased during and increased after ending adjuvant chemotherapy. Post hoc analyses are shown in Supplementary Table 1.

**Table 3:** Informal caregivers’ course of burden, distress, health-related quality of life, marital satisfaction, fatigue, and self-esteem

	Population norm scores	T0	T1	T2	Sign
	Mean (SD)	Mean (SD)	Mean (SD)	Mean (SD)	*p* value
Burden (SPPIC) [14, 15]	a	2.90 (2.44)	2.82 (2.38)	2.04 (2.25)	0.014*
Distress (HADS) [27]	8.4 (6.3)	8.96 (6.91)	7.57 (6.32)	6.64 (7.15)	0.008*
Health-related quality of life (RAND 36) [19]					
Physical functioning	81.90 (23.20)	91.04 (10.47)	90.94 (13.90)	87.81 (16.24)	0.153
Role limitations due to physical health	79.40 (35.50)	79.89 (23.20)	87.50 (22.82)	84.78 (30.95)	0.253
Role limitations due to emotional problems	84.10 (32.30)	63.88 (41.73)	81.25 (34.32)	86.11 (30.62)	< 0.0001*
Vitality	67.40 (19.90)	67.29 (14.73)	71.98 (15.04)	71.88 (20.39)	0.067
Mental health	76.80 (18.40)	73.00 (14.16)	77.08 (12.83)	78.33 (14.41)	0.015*
Social functioning	86.90 (20.50)	80.00 (20.52)	84.75 (18.09)	87.00 (15.14)	0.022*
Pain	79.50 (25.60)	92.00 (12.98)	89.63 (15.63)	88.30 (17.17)	0.173
General health	72.70 (22.70)	74.56 (16.68)	75.56 (16.42)	73.33 (20.83)	0.430
Marital satisfaction (MMQ) [22, 28]	13.58 (10.79)	9.76 (9.94)	10.37 (10.80)	11.66 (11.53)	0.189
Social support discrepancies (SSL-D) [21]	43.6 (10.3)	41.18 (9.09)	38.38 (5.68)	39.06 (6.63)	0.037*
Fatigue (AFQ) [24]	b	10.02 (2.65)	10.49 (6.30)	10.55 (7.26)	0.748
Self-esteem (CRA) [29]	4.19 (0.41)	4.32 (0.54)	4.32 (0.49)	4.23 (0.48)	0.181

*p values represent significant changes in mean scores over time; references given in this table refer to population norm scores. *SPPIC* Self-Perceived Pressure of Informal Care, *HADS* Hospital Anxiety and Depression Scale, *MMQ* Maudsley Marital Questionnaire, *SSL-D* Social Support List – Discrepancies, *AFQ* Abbreviated Fatigue Questionnaire, *CRA* Caregiver Reaction Assessment, *T0* baseline, *T1* between 2nd and 3rd cycles of adjuvant chemotherapy, *T2* 3 months after ending adjuvant chemotherapy

^a^Scores for levels of burden: 0–3 low, 4–6 moderate, 7–9 high

^b^Scores for level of fatigue: low = 4, below average = 4, average = 5–8, above average = 9–14, high ≥ 15

Table 4 displays patients’ mean scores for health-related quality of life, marital satisfaction, discrepancies in social needs, and fatigue over time. Mean patient distress (*F*[1.771,79.706] = 5.224; *p* = 0.010), role physical limitations (*F*[2,94] = 9.551; *p* < 0.0001), vitality (*F*[2,96] = 5.295; *p* = 0.007), social functioning (*F*[2,96] = 9.157; *p* < 0.0001), general health (*F*[2,92] = 6.672; *p* = 0.002), marital satisfaction (*F*[2,54] = 5.395; *p* = 0.007), and fatigue (*F*[2,92] = 11.393; *p* < 0.0001) changed significantly over time. Post hoc analyses revealed an overall decrease of distress and increase in role physical limitations and social functioning. Vitality and general health decreased during and increased after ending adjuvant chemotherapy. Marital satisfaction decreased over time. Fatigue increased during and decreased after ending adjuvant chemotherapy. Post hoc analyses are shown in Supplementary Table 2.

**Table 4:** Patient course of distress, health-related quality of life, marital satisfaction, fatigue, and self-esteem

	Population norm scores	T0	T1	T2	Significant difference over time
	Mean (SD)	Mean (SD)	Mean (SD)	Mean (SD)	*p* value
Distress (HADS) [27]	8.4 (6.3)	7.22 (6.04)	7.37 (5.41)	5.54 (4.31)	0.010*
Health-related quality of life (RAND 36) [19]					
Physical functioning	81.90 (23.20)	76.35 (19.76)	76.15 (20.78)	79.52 (20.85)	0.168
Role limitations due to physical health	79.40 (35.50)	31.77 (40.52)	33.33 (38.71)	59.36 (45.44)	< 0.0001*
Role limitations due to emotional problems	84.10 (32.30)	65.93 (44.66)	74.81 (40.92)	85.19 (33.75)	0.061
Vitality	67.40 (19.90)	64.59 (17.61)	58.16 (18.33)	64.90 (19.59)	0.007*
Mental health	76.80 (18.40)	79.92 (14.64)	70.49 (14.33)	82.45 (11.07)	0.344
Social functioning	86.90 (20.50)	68.37 (19.45)	72.45 (20.41)	82.14 (19.76)	< 0.0001*
Pain	79.50 (25.60)	74.90 (23.06)	74.82 (22.63)	80.42 (20.56)	0.285
General health	72.70 (22.70)	63.51 (19.33)	60.00 (18.44)	68.62 (20.50)	0.002*
Marital satisfaction (MMQ) [28]	13.58 (10.79)	5.79 (6.85)	6.86 (8.37)	9.00 (9.58)	0.007*
Social support discrepancies (SSL-D) [21]	43.2 (10.7)	36.86 (4.90)	36.19 (3.03)	36.06 (3.18)	0.445
Fatigue (AFQ) [24]	a	12.36 (6.61)	16.21 (7.23)	12.85 (6.37)	< 0.0001*

*p values represent changes in mean scores over time; references given in this table refer to population norm scores. *HADS* Hospital Anxiety and Depression Scale, *MMQ* Maudsley Marital Questionnaire, *SSL-D* Social Support List – Discrepancies, *AFQ* Abbreviated Fatigue Questionnaire, *T0* baseline, *T1* between 2nd and 3rd cycles of adjuvant chemotherapy, *T2* 3 months after ending adjuvant chemotherapy

^a^Scores for level of fatigue: low = 4, below average = 5–12, average = 13–21, above average = 22–27, high ≥ 28

### Clinically relevant levels of informal caregiver burden and distress

Moderate or high levels of burden of informal caregivers were found in 17.2% (*n* = 10/58) and 12.1% (*n* = 7/58) at baseline, 19.3% (*n* = 11/57) and 10.5% (*n* = 6/57) at T1, and 12% (*n* = 6/50) and 8% (*n* = 4/50) at T2, respectively. A HADS total score exceeding the cutoff for clinically relevant distress was found in 26.7% (*n* = 16/60) at baseline, 22.8% (*n* = 13/57) at T1, and 18.8% (*n* = 9/48) at T2. Clinically relevant levels of informal caregiver depressive symptoms (HADS-D) were 15% at baseline, 10.5% at T1, and 12.2% at T2. Clinically relevant levels of informal caregiver anxiety (HADS-A) were 21.7% at baseline, 21.1% at T1, and 12.2% at T2. In patients, 22.4% (*n* = 13/58) exceeded the cutoff at baseline for clinically relevant levels of distress, 31.5% (*n* = 17/54) at T1, and 18.0% (*n* = 9/50) at T2. Clinically relevant levels of patient depression were 12.1% at baseline, 16.1% at T1, and 7.8% at T2. Clinically relevant levels of patient anxiety were 17.2% at baseline, 13.0% at T1, and 7.8% at T2. There was no significant difference between the proportions of informal caregivers (*p* = 0.289) or patients (*p* = 0.508) with clinically relevant levels of distress at baseline and T2.

### Predictors of informal caregiver distress and burden at T1 and T2

Informal caregiver gender, age, burden, distress, fatigue, and patient distress at baseline predicted informal caregiver self-perceived burden at T1 (*F*[6,53] = 4.493, *p* = 0.001, *R*^2^ = 0.365) and T2 (*F*[6,46] = 4.523, *p* < 0.001, *R*^2^ = 0.404). Only informal caregiver burden at baseline added significantly to the prediction at T1 (*p* = 0.002) and T2 (*p* = 0.002). The multivariate regression model was also used to predict distress at T1 (*F*[6,53] = 12.305, *p* < 0.0001, *R*^2^ = 0.611) and T2 (*F*[6,44] = 7.204, *p* < 0.0001, *R*^2^ = 0.532). Only baseline informal caregiver distress (*p* < 0.001) added significantly to the prediction at T1 (*p* < 0.001) and T2 (*p* = 0.001). Details are displayed in Table 5.

**Table 5:** Multivariate regression analyses to explore associations with informal caregiver burden and distress during and after patients’ treatment with adjuvant chemotherapy

	Burden T1		Burden T2		Distress T1		Distress T2	
	Beta	*p* value	Beta	*p* value	Beta	*p* value	Beta	*p* value
Informal caregiver characteristics								
Age	0.021	0.862	− 0.134	0.290	− 0.028	0.761	− 0.066	0.876
Gender	− 0.082	0.497	− 0.032	0.799	− 0.008	0.931	0.042	0.819
Burden T0	0.440	0.002*	0.478	0.002*	− 0.011	0.917	0.002	0.988
Distress T0	0.294	0.065	0.100	0.533	0.716	0.000*	0.544	0.001*
Fatigue T0	− 0.056	0.720	0.115	0.472	0.119	0.335	0.275	0.063
Patient characteristics								
Distress T0	0.014	0.909	0.037	0.777	− 0.110	0.258	− 0.105	0.395
*R*^2^	37%		40%		61%		53%	

### Interaction between informal caregivers and patients

Generally, informal caregivers reported higher distress levels (T0 mean 8.47 (SD 6.83); T1 mean 7.42 (SD 6.34); T2 mean 6.50 (SD 7.14)) compared with patients (T0 mean 7.02 (SD 5.80); T1 mean 7.20 (SD 5.93); T2 mean 5.56 (SD 4.69)), but these differences did not reach statistical significance (T0 mean difference 1.45, *p* = 0.217; T1 mean difference 0.22, *p* = 0.853; T2 mean difference 0.94, *p* = 0.445). We did not find significant correlations between informal caregiver and patient distress at baseline (*r* = 0.134, *p* = 0.315), T1 (*r* = 0.263, *p* = 0.054), or T2 (*r* = 0.121, *p* = 0.424). In addition, informal caregiver burden and patient distress did not correlate significantly at T1 (*r* = 0.209, *p* = 0.129) or T2 (*r* = 0.205, *p* = 0.167). However, at baseline, informal caregiver burden was significantly, but weakly correlated with patient distress (*r* = 0.261, *p* = 0.05).

## Discussion

The present longitudinal study explored the course of informal caregiver well-being, clinically relevant levels of informal caregiver burden and distress, and baseline risk factors for higher levels of burden and distress during and after a patients’ adjuvant chemotherapy for colon cancer. We found that informal caregivers seem to report more mental problems before and during chemotherapy, whereas patients report more physical problems. Additionally, 20–30% of informal caregivers report relevant levels of burden and distress, between, before, during, and after a patients’ treatment. Baseline burden and distress are risk factors for burden and distress during and after a patients’ adjuvant treatment.

Informal caregivers reported more mental than physical problems after a patient’s cancer diagnosis and during treatment, and social functioning seemed to be influenced negatively. This was also found in another study on the impact of colorectal cancer on patients and their partners [30]. This study of Traa et al. showed that both partners and patients suffer mentally and in social functioning, which is in line with our findings. Furthermore, a study of Law et al. showed that informal caregivers’ social functioning changed due to fear of burdening others, and when informal caregivers do get support, this support is perceived insufficient and not what they need on that moment [31]. Also, informal caregivers report that family and friends become avoidant in their contact with the patient and informal caregiver, which challenges their social interactions [32]. In contrast to the findings of Traa et al. who found a stabilization in mental well-being and social functioning, we observed an improvement over time. A possible explanation could be that in the study of Traa et al., 60% had an colostomy after surgery compared with only 13% in our study. Having a colostomy is known to cause distress for the patient and their informal caregiver and may impact social activities and increase social isolation [32–35].

Before starting adjuvant chemotherapy, almost 30% of informal caregivers reported moderate-to-high levels of burden and clinically relevant distress, which decreased to 20% 3 months after the end of adjuvant treatment. Ohlsson-Nevo et al. pointed out that partners’ lives are turned upside down after colorectal cancer diagnosis, being confronted with how fragile life is [33]. They had to deal with new and other unwanted responsibilities at home that they felt compelled to fulfill [33]. Northouse et al. found that during patients’ treatment for cancer, informal caregivers experienced worries about the effectiveness of the treatment, accompanied by difficulties managing side effects [6]. For informal caregivers who still experienced burden and distress after the patient’s completion of adjuvant chemotherapy, a possible contributing factor could be the ongoing treatment toxicity, which was reported by 61% of the patients in our study. Although toxicity can diminish over time, there is a group of patients for which the toxicity, in particular peripheral neuropathy, remains a limiting factor in a patients’ life [36], and thereby also influences the life of their significant others. Fortunately, there are new insights for treating stage III colon cancer, namely a shift from 6 to 3 months CAPOX, which is non-inferior in terms of survival and induces less toxicity [36]. Our study was performed when these data were not available yet. Since the number of cycles will be reduced and thereby also the associated cumulative toxicity, this may ultimately also positively impact the informal caregiver [36]. Another contributing factor for ongoing burden and distress can be fear of cancer recurrence. This is found in patients, and van de Wal et al. found that partners report the same levels of fear of recurrence as well. [37]

When thinking of ways to support informal caregivers in need, it could be particularly helpful to predict who is in need of support and when. In our study, we found that informal caregiver burden and distress at baseline predicted informal caregiver burden and distress both during adjuvant chemotherapy and 3 months after completion of treatment. Jansen et al. reported that informal caregivers of patients with different types of cancer with higher baseline levels of burden remained burdened over the following years [38]. It is possible that informal caregivers who remain burdened and distressed have more difficulties coping, as negative coping skills are associated with higher levels of burden and distress [39]. Further research with ongoing assessment of burden and distress due to the dynamic nature of these constructs, with a focus on what causes and maintains burden and distress, is recommended. Especially it is known that long-term burden and distress cause serious general health problems [7]. Additionally, our study shows that informal caregivers’ distress exceeds patients’ distress. This finding is consistent with those of other studies [6, 30]. Based on these findings, we recommend to pay attention and offer support when informal caregiver burden and distress are observed before starting adjuvant chemotherapy, or even earlier, shortly after diagnosis. More specifically, repeated assessment of informal caregiver needs for more practical support or other informational services from care professionals is recommended. Importantly, the management of treatment side effects deserves special attention as 60% of informal caregivers report that their well-being is negatively influenced by patients’ side effects during treatment. Additionally, based on our inquiry of informal caregiver needs, the general practitioner may be particularly well-suited for providing support to the informal caregiver, as leading practitioner in informal caregiver care.

Our exploratory longitudinal study adds significantly to the scarce literature on informal caregiver burden and distress during adjuvant treatment of colon cancer. However, it is important to take into account several limitations. First, the sample size is relatively small which prevented us from studying additional predictors of informal caregiver burden and distress, such as the influence of a patient’s colostomy. Second, patients and informal caregivers who declined participation in this study often refused because participation was perceived too burdensome. This may limit the generalizability of our findings and our conclusion on burden and distress to the larger population of informal caregivers, and our findings may be an underestimation. Third, although we assessed statistical significance of changes in informal caregiver well-being over time, we were unable to determine clinical relevance due to the lack of established minimally clinically important differences (MCID) for the measures that we used. Future research assessing MCIDs for among informal caregiver population is warranted.

In conclusion, before and during adjuvant chemotherapy, informal caregivers report more mental problems whereas patients report more physical problems. When informal caregivers and patients experience problems before start of adjuvant chemotherapy, problems seem to improve over time. Nevertheless, approximately 20% of informal caregivers remain burdened and distressed after patients’ end of treatment, and remarkably informal caregivers’ distress exceeds patients’ distress. Additionally, informal caregivers’ baseline burden and distress seem to be risk factors for ongoing burden and distress after treatment. Therefore, it is of great importance to identify burden and distress among informal caregivers of patients treated with adjuvant chemotherapy for colon cancer and offer them support according to their needs.

## Electronic supplementary material

Supplementary Table 1 (XLSX 11 kb)

Supplementary Table 2 (XLSX 11 kb)

## References

[1] Union for International Cancer Control - GLOBOCAN (2018) https://www.uicc.org/news/new-global-cancer-data-globocan-2018

[2] Incidentie darmkanker www.cijfersoverkanker.nl

[3] Labianca R, Nordlinger B, Beretta GD, Mosconi S, Mandala M, Cervantes A, Arnold D, Group EGW (2013). Early colon cancer: ESMO Clinical Practice Guidelines for diagnosis, treatment and follow-up. Ann Oncol.

[4] Shah MA, Renfro LA, Allegra CJ, Andre T, de Gramont A, Schmoll HJ, Haller DG, Alberts SR, Yothers G, Sargent DJ (2016). Impact of patient factors on recurrence risk and time dependency of oxaliplatin benefit in patients with colon cancer: analysis from modern-era adjuvant studies in the Adjuvant Colon Cancer End Points (ACCENT) database. J Clin Oncol.

[5] Andre T, Boni C, Navarro M, Tabernero J, Hickish T, Topham C, Bonetti A, Clingan P, Bridgewater J, Rivera F, de Gramont A (2009). Improved overall survival with oxaliplatin, fluorouracil, and leucovorin as adjuvant treatment in stage II or III colon cancer in the MOSAIC trial. J Clin Oncol.

[6] Northouse LL, Katapodi MC, Schafenacker AM, Weiss D (2012). The impact of caregiving on the psychological well-being of family caregivers and cancer patients. Semin Oncol Nurs.

[7] Northouse L, Williams AL, Given B, McCorkle R (2012). Psychosocial care for family caregivers of patients with cancer. J Clin Oncol.

[8] Applebaum AJ, Breitbart W (2012). Care for the cancer caregiver: a systematic review. Palliat Support Care.

[9] Adelman RD, Tmanova LL, Delgado D, Dion S, Lachs MS (2014). Caregiver burden: a clinical review. JAMA.

[10] National Cancer Institute - definition of distress. https://www.cancer.gov/publications/dictionaries/cancer-terms/def/distress

[11] Kim Y, van Ryn M, Jensen RE, Griffin JM, Potosky A, Rowland J (2014). Effects of gender and depressive symptoms on quality of life among colorectal and lung cancer patients and their family caregivers. Psycho-oncology..

[12] Northouse LL, Mood D, Templin T, Mellon S, George T (2000). Couples’ patterns of adjustment to colon cancer. Soc Sci Med.

[13] Sangha O, Stucki G, Liang MH, Fossel AH, Katz JN (2003). The Self-Administered Comorbidity Questionnaire: a new method to assess comorbidity for clinical and health services research. Arthritis Rheum.

[14] Pot AM, van Dyck R, Deeg DJ (1995). Perceived stress caused by informal caregiving. Construction of a scale. Tijdschr Gerontol Geriatr.

[15] Haj Mohammad N, Walter AW, van Oijen MG, Hulshof MC, Bergman JJ, Anderegg MC, van Berge Henegouwen MI, Henselmans I, Sprangers MA, van Laarhoven HW (2015). Burden of spousal caregivers of stage II and III esophageal cancer survivors 3 years after treatment with curative intent. Support Care Cancer.

[16] Langenberg S, Reyners AKL, Wymenga ANM, Sieling GCM, Veldhoven CMM, van Herpen CML, Prins JB, van der Graaf WTA (2019). Caregivers of patients receiving long-term treatment with a tyrosine kinase inhibitor (TKI) for gastrointestinal stromal tumour (GIST): a cross-sectional assessment of their distress and burden. Acta Oncol.

[17] Langenberg S, van Herpen CML, van Opstal CCM, Wymenga ANM, van der Graaf WTA, Prins JB (2019). Caregivers’ burden and fatigue during and after patients’ treatment with concomitant chemoradiotherapy for locally advanced head and neck cancer: a prospective, observational pilot study. Support Care Cancer.

[18] Zigmond AS, Snaith RP (1983). The hospital anxiety and depression scale. Acta Psychiatr Scand.

[19] VanderZee KI, Sanderman R, Heyink JW, de Haes H (1996). Psychometric qualities of the RAND 36-Item Health Survey 1.0: a multidimensional measure of general health status. Int J Behav Med.

[20] Bridges KR, Sanderman R, van Sonderen E (2002). An English language version of the social support list: preliminary reliability. Psychol Rep.

[21] VanSonderen E (2012) Het meten van sociale steun met de Sociale Steun Lijst - Interacties (SSL-I) en Sociale Steun Lijst - Discrepanties (SSL-D): een handleiding. Tweede herziene druk

[22] Arrindell WA, Schaap C (1985). The Maudsley Marital Questionnaire (MMQ): an extension of its construct validity. Br J Psychiatry.

[23] Bleijenberg G, Knoop H, Gielissen M (2009) [‘Abbreviated Fatigue Questionniare to establish the severity of chronic fatigue’] Bijblijven (Tijdschrift Praktische Huisartsgeneeskunde) (1):19–21

[24] Alberts M, Smets EM, Vercoulen JH, Garssen B, Bleijenberg G (1997). ‘Abbreviated fatigue questionnaire’: a practical tool in the classification of fatigue. Ned Tijdschr Geneeskd.

[25] Given CW, Given B, Stommel M, Collins C, King S, Franklin S (1992). The caregiver reaction assessment (CRA) for caregivers to persons with chronic physical and mental impairments. Res Nurs Health.

[26] Vodermaier A, Millman RD (2011). Accuracy of the Hospital Anxiety and Depression Scale as a screening tool in cancer patients: a systematic review and meta-analysis. Support Care Cancer.

[27] Spinhoven P, Ormel J, Sloekers PP, Kempen GI, Speckens AE, Van Hemert AM (1997). A validation study of the Hospital Anxiety and Depression Scale (HADS) in different groups of Dutch subjects. Psychol Med.

[28] Joseph O, Alfons V, Rob S (2007). Further validation of the Maudsley Marital Questionnaire (MMQ). Psychol Health Med.

[29] Nijboer C, Triemstra M, Tempelaar R, Sanderman R, van den Bos GA (1999). Measuring both negative and positive reactions to giving care to cancer patients: psychometric qualities of the Caregiver Reaction Assessment (CRA). Soc Sci Med.

[30] Traa MJ, Braeken J, De Vries J, Roukema JA, Orsini RG, Den Oudsten BL (2015). Evaluating quality of life and response shift from a couple-based perspective: a study among patients with colorectal cancer and their partners. Qual Life Res.

[31] Law E, Levesque JV, Lambert S, Girgis A (2018). The “sphere of care”: a qualitative study of colorectal cancer patient and caregiver experiences of support within the cancer treatment setting. PLoS One.

[32] Cotrim H, Pereira G (2008). Impact of colorectal cancer on patient and family: implications for care. Eur J Oncol Nurs.

[33] Ohlsson-Nevo E, Andershed B, Nilsson U, Anderzen-Carlsson A (2012). Life is back to normal and yet not – partners’ and patient’s experiences of life of the first year after colorectal cancer surgery. J Clin Nurs.

[34] Maguire R, Hanly P, Hyland P, Sharp L (2018) Understanding burden in caregivers of colorectal cancer survivors: what role do patient and caregiver factors play? Eur J Cancer Care (Engl) 27 (1). doi:10.1111/ecc.12527 10.1111/ecc.12527 27271848

[35] Nijboer C, Triemstra M, Tempelaar R, Sanderman R, van den Bos GA (1999). Determinants of caregiving experiences and mental health of partners of cancer patients. Cancer.

[36] Grothey A, Sobrero AF, Shields AF, Yoshino T, Paul J, Taieb J, Souglakos J, Shi Q, Kerr R, Labianca R, Meyerhardt JA, Vernerey D, Yamanaka T, Boukovinas I, Meyers JP, Renfro LA, Niedzwiecki D, Watanabe T, Torri V, Saunders M, Sargent DJ, Andre T, Iveson T (2018). Duration of adjuvant chemotherapy for stage III colon cancer. N Engl J Med.

[37] van de Wal M, Langenberg S, Gielissen M, Thewes B, van Oort I, Prins J (2017). Fear of cancer recurrence: a significant concern among partners of prostate cancer survivors. Psycho-oncology.

[38] Jansen L, Dauphin S, De Burghgraeve T, Schoenmakers B, Buntinx F, van den Akker M (2019) Caregiver burden: an increasing problem related to an aging cancer population. J Health Psychol:1359105319893019. 10.1177/1359105319893019 10.1177/1359105319893019 31814462

[39] Palacio C, Krikorian A, Limonero JT (2018). The influence of psychological factors on the burden of caregivers of patients with advanced cancer: resiliency and caregiver burden. Palliat Support Care.

